# Variations in Content and Extractability of Durum Wheat (*Triticum turgidum L. var durum*) Arabinoxylans Associated with Genetic and Environmental Factors

**DOI:** 10.3390/ijms12074536

**Published:** 2011-07-15

**Authors:** Roberto Ciccoritti, Giulia Scalfati, Alessandro Cammerata, Daniela Sgrulletta

**Affiliations:** Research Unit for Cereal Quality, CRA (Agricultural Research Council), Via Cassia 176, 00191 Rome, Italy; E-Mails: ciccorittiroberto@libero.it (R.C.); giulia.scalfati@entecra.it (G.S.); alessandro.cammerata@entecra.it (A.C.)

**Keywords:** durum wheat, total and water extractable arabinoxylans, functional foods, high fiber pasta

## Abstract

Arabinoxylans (AX) represent the most abundant components of non-starch polysaccharides in wheat, constituting about 70% of cell wall polysaccharides. An important property of AX is their ability to form highly viscous water solutions; this peculiarity has a significant impact on the technological characteristics of wheat and determines the physiologically positive influence in consumption. Durum wheat (*Triticum turgidum L. var durum*), the raw material for pasta production, is one of the most important crops in Italy. As part of a large project aimed at improving durum wheat quality, the characterization of the nutritional and technological aspects of whole grains was considered. Particular attention was addressed to identify the best suited genotypes for the production of innovative types of pasta with enhanced functional and organoleptic properties. The objective of the present study was to investigate the genetic variability of AX by examining a group of durum wheat genotypes collected at two localities in Italy for two consecutive years. The environmental influence on AX content and extractability was also evaluated. Variability in the AX fraction contents was observed; the results indicated that AX fractions of durum wheat grain can be affected by the genotype and environment characteristics and the different contribution of genotype and environment to total variation was evidenced. The genotype × environment (G × E) interaction was significant for all examined traits, the variations due to G × E being lower than that of genotype or environment. The data and the statistical analysis allowed identification of the Italian durum wheat varieties that were consistently higher in total arabinoxilans; in addition, principal component analysis biplots illustrated that for arabinoxylan fractions some varieties responded differently in various environment climatic conditions.

## 1. Introduction

A large amount of research underlined the significant role that the dietary fiber components play in improving human health and emphasized that a high intake of fiber and of whole cereal grains is a key component of a healthy dietary pattern [1,2]. Following the new trend for healthy foods, cereal whole grains have received much attention, especially in order to minimise losses of healthy constituents of grains during processing [3–6]. In fact, most bioactive components (vitamins, minerals, antioxidants, phytoestrogens and fiber, soluble and insoluble) are contained in the outer part of the kernel or in the germ, that are removed during the traditional milling process [7]. Therefore, the consumption of whole grain foods could allow an adequate intake of dietary fiber. Several studies have demonstrated its role in reducing the risk of many diseases (diabetes, cardiovascular disease, certain cancers) and in improving gut health, recommending that its intake be increased in the human diet [8–12]. Moreover, specific healthy effects on chronic diseases have been associated with different fiber components; soluble fractions have, in particular, attracted much attention for their property to increase food viscosity by determining many of the actual physiological effects. Viscous β-glucans were proved to reduce cholesterol level and to affect blood glucose and insulin response in humans [9]; similar effects were also suggested for arabinoxylans (AX), the most abundant polysaccharide constituents of the cell wall in wheat (approximately 70%) [13]. AX are classified as water extractable (WEAX) and water unextractable (WUAX) fractions. Water extractability depends on the structural features of the polymer chain (degree of arabinose substitution, ferulic acid cross linking and degree of xylan polymerization) and, also, on covalent linkage to other cell-wall polymers [14]. The most relevant structural feature of AX is the main chain of (1,4)-β-linked D-xylose residues to which l-arabinofuranose is *α*-linked to the hydroxyl group on C-2 or C-3 (or both) of some of the D-xylosyl residues along the xylan backbone, with some ferulic acid esterified to arabinose on O_5_ [15]. The AX, heterogeneous and complex molecules, appear to play the major effect both on technological and nutritional properties of wheat through their high water-holding capacity and their ability to form highly viscous water solutions [16–18]. Numerous studies investigated the influence of genotype and growing environment on the AX contents of the wheat grain (*T. aestivum*). For total AX, a range between 5.5 and 7.8% dry weight (d.w.) was referred for wheat grain by Saulnier *et al*. [19]. Large variations in content and structural features of WEAX in wheat flour of French varieties and the large impact of genotype on AX structure were evidenced by Ordaz-Ortiz and Saulnier [20]. Genetic control of AX structural variability and genetic differences among genotypes for arabinoxylan fractions were previously indicated [14,21,22]. Moreover, Finnie *et al*. [23] showed that, in soft wheat, the variations in AX content were primarily due to genotype, the environment having a secondary effect. In contrast, the studies on the AX in durum wheat grains are scarce. Total arabinoxylan (TOAX) contents between 4–6% dry matter (d.m.) were indicated by Lempereur *et al*. [24] by analyzing the whole grain of five French durum wheat varieties and by Gebruers *et al*. [25] that within the HEALTHGRAIN project examined a set of European durum wheat varieties. Turner *et al*. [26] presented a wider range of water extractable arabinopolymer contents (0.59–7.21 μg/mg^−1^) in Australian durum genotypes than in bread wheat cultivars and indicated their positive influence on pasta quality through a significant reduction of pasta stickiness.

Durum wheat (*Triticum turgidum L. var durum*) is one of the most important crops in Italy and the exclusive raw material for pasta production for the Italian law (Italian law No. 580, 1967). It is well known that the intrinsic characteristics of raw material as well as the technological process conditions are the main factors affecting the traditional pasta cooking quality traits [27,28]. Over the last decade there has been an increased availability on the market of high-fiber pasta produced by enriching semolina or by directly processed durum wheat grains without removing the bran [29]. As a part of a project aimed at improving durum wheat quality, the characterization of the nutritional and technological aspects of whole grains was considered. Particular attention was addressed to identify the best suited genotypes for the production of innovative types of pasta with enhanced organoleptic characteristics and high functional properties. The aim of the present work was to investigate the AX content in a wide range of Italian durum wheat cultivars grown in two localities of Italy (Jesi, Central-Northern Italy, and Foggia, Southern Italy) for two consecutive years (2008–2009 and 2009–2010) and to evaluate the effects of genetic and environmental variations on the final AX level as well as on its extractability in whole durum wheat grains.

## 2. Results and Discussion

### 2.1. Grain Characteristics and Relationships among Grain Traits

Table 1 shows the mean value and the standard deviation across thirty cultivars for all the traits in each of the four environments. On average, the environments presented variations in grain weight, whereas the other physical characteristics did not differ significantly. The hardness index values indicated that the durum wheat samples exhibited the natural variability in very hard kernel texture for each of the considered environments. As expected, the protein concentration was shown to be highly sensitive to the growing site. In fact, in the durum wheat varieties grown in Jesi the protein content measured was greater than that grown in Foggia in both the years; these differences likely reflected the major availability of nitrogen fertilizer during grain filling and different soil fertility. The average TOAX contents of the four environments ranged from 4.5 to 4.8% d.m., Jesi09 having lower WUAX content than the other environments. As indicated in the experimental section the WEAX amount was calculated by difference (TOAX–WUAX); on average, among the four environments the range in WEAX was very limited.

There were significant correlations between protein content and the physical characteristics of the grain (*r* = 0.317 *p* ≤ 0.01; *r* = 0.379 *p* ≤ 0.001; *r* = 0.225 *p* ≤ 0.05, with hardness index, grain weight and diameter, respectively, *n* = 76). The reported relationships were found to be significant both in the single environments and in the whole trial. No correlations between AX fractions and hardness index were found, in agreement with Bettge and Morris [30] who suggested a minimal role of pentosans in modifying hard wheat grain hardness and with the results of Li *et al*. [22] in hard and spring wheat.

### 2.2. ANOVA

**Figure 1:**
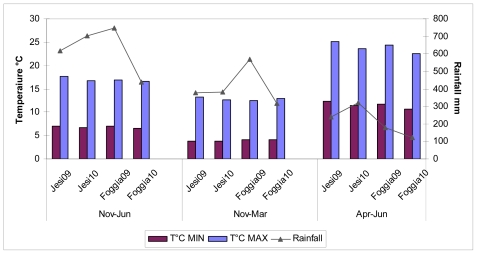
Average temperatures (°C) and rainfall (mm) in the growing environments: Foggia09, Foggia10, Jesi09 and Jesi10 in three periods of durum wheat growth.

A group of 19 durum wheat cultivars common to the four environments was considered for ANOVA of AX. The results presented in Table 2 evidenced highly significant genotypic and environmental effects and a significant genotype x environment interaction for all the AX components. However, as previously evidenced by Finnie *et al*. [23] and by Gebruers *et al*. [31] in wheat whole meal, the contribution of this interaction to the total variability was considerably lower than that of genotype or environment. Variety had the greatest influence on the WUAX content in durum wheat samples and was important for TOAX. The results of this study indicated that great variations could be ascribed to different environmental conditions, environment being the dominant factor contributing to total variations of AX fraction content. These results are generally consistent with the results of Lempereur *et al*. [24] who evidenced the influence both of cultivar and environment for TOAX and WEAX fractions in durum wheat grain. As illustrated in Figure 1, different climatic conditions were registered in the four trials, especially in relation to max temperatures in the last phase of grain filling and total rainfall (see also experimental section). On average, the highest TOAX values were found in Jesi10 (4.9 ± 0.39% d.m.) and Foggia09 (4.6 ± 0.22% d.m.), two experiments with the highest amounts of rain. In general, the lowest values of WUAX were evidenced in Jesi09 (3.8 ± 0.23% d.m.). These results were in agreement with the observations of Li *et al*. [22], who detected the highest level of TOAX in the environments where higher amounts of rainfall occurred. The influence of water availability on AX accumulation was discussed by Dornez *et al*. [32], who in wheat grain evidenced significantly higher amount of WEAX during the rainy years. Other studies on common wheat have also reported environmental effects on the amount of AX fractions [31–34], the impact of genotype being more evident on structural AX changes [14]. Gebruers *et al*. [31] observed different variation in relation to environment climatic characteristics and concluded that the total variation in the AX levels was determined by the interaction with other factors such as agronomical input and soil type.

### 2.3. Varietal Variability for AX Fractions

Varietal comparison of AX fractions based on Duncan’s test are reported in Table 3. Significant variation existed among the 19 durum wheat varieties for AX fractions. The mean values of genotypes across environments ranged from 4.3 to 5.2% d.m. and from 3.7 to 4.6% d.m. for TOAX and WUAX, respectively. The TOAX range was comparable to the results obtained by Lempereur *et al*. [24] with a different analysis method.

The results showed that, with regard to TOAX and WUAX contents, some cultivars were significantly superior to the others: Trionfo, followed by Alemanno, presented the highest means for TOAX and WUAX fractions, having consistently the highest TOAX content in three of the four environments analyzed (data not shown). Among the considered durum wheat varieties the WEAX range varied from 0.5 to 0.9% d.m., Creso exhibiting the highest content. Cultivars Tirex and Saragolla were the poorest for TOAX and WUAX. The WUAX appeared to vary within a larger range for some varieties in the various environments; Trionfo, Tirex, Simeto, Ciccio, Arnacoris and Imhopet presented particularly higher variability in comparison with the TOAX. The ratio WEAX/WUAX, considered a measure of extractability [24], varied from 10.9 (Arnacoris) to 23.4% (Creso) the mean value being 16.3%. As found by Lempereur *et al*. [24], the extractability of AX in this study also appeared to be independent of the TOAX content (*data not reported*).

### 2.4. AX Fraction Variability in Monosaccharide Composition

The ratio between arabinose (A) and xylose (X) obtained with the AX fractions was also calculated, the measurement of this ratio partially characterizing the structure of the AX [14]. In addition, Martinant *et al*. [35] found a strong negative correlation between A/X ratio and relative viscosity measurements of the flour water extracts and, following the suggestions of Ordaz-Ortiz and Saulnier [20], variations in the A/X ratio could likely indicate different degrees of arabinose substitution in the different AX fractions. Considering the mean values across environments of the 19 durum wheat varieties, WUAX had A/X ratio values ranged from 0.71 to 0.82 (mean value: 0.78 ± 0.027; *data not shown*). Similar values were obtained by Barron *et al*. [36] in whole grains of two soft wheat cultivars. WUAX appeared mainly composed by arabinose and xylose (mean values: 38.1% and 49.0%, respectively). Small amounts of mannose were also found. As compared with TOAX, WUAX had higher level of arabinose and xylose. No significant differences in the AX fraction composition were found among the durum wheat varieties. Under the measure conditions of this study only some indications were drawn for the WEAX fraction composition, arabinose and xylose still remaining the main sugars (26.4 and 34.7%, respectively). The compositional variations likely accounted for the localization of AX fractions in the whole grain, WUAX deriving from the endosperm cell walls but mainly from the outer layer of the grain. Some other changes in monosaccharide composition likely reflected structural differences of cell wall matrix and were associated with the presence of WEAX only in the starchy endosperm (*data not reported*). As shown by Martinant *et al*. [35], TOAX content was logically negatively related to xylose percentage (*r* = −0.696, *p* ≤ 0.001), but, in contrast with the results of Martinant *et al*. [35], this ratio was positively related to arabinose percentage (*r* = 0.823, *p* ≤ 0.001).

### 2.5. Principal Component Analysis (PCA)

**Figure 2:**
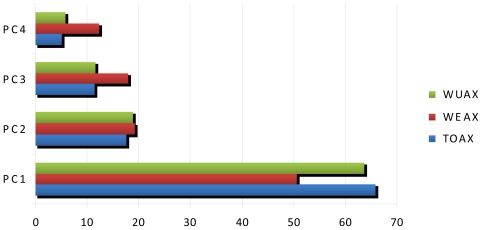
Composition of variance, explained by different principal components: Principal component (PC)1, PC2, PC3 and PC4, for AX fractions of durum wheat (total, TOAX, water extractable, WEAX and water unextractable, WUAX).

**Figure 3:**
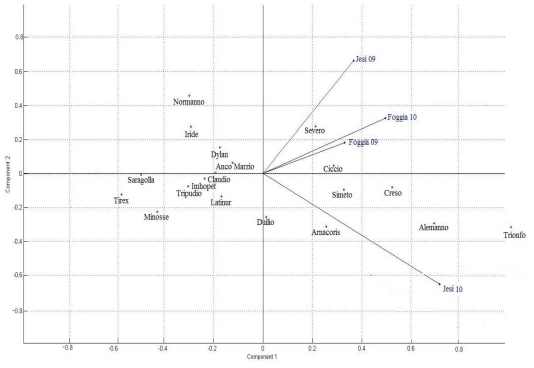
Genotype × environment biplot from principal component analysis for TOAX (total arabinoxylans).

**Figure 4:**
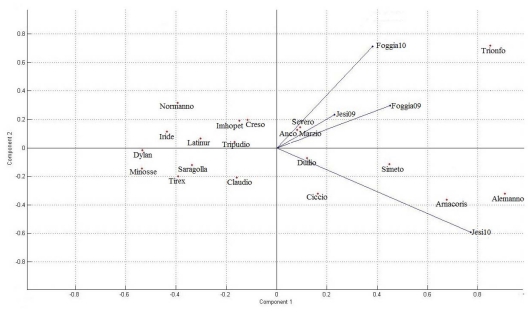
Genotype × environment biplot from principal component analysis for WUAX (water unextractable arabinoxylans).

As previously discussed, genotype and environment strongly affected AX concentrations of durum wheat whole grain in this study, the variations due to G × E interaction being lower than that of genotype or environment. The results, however, evidenced that the varieties did not respond similarly to different environmental conditions and, in agreement with the results of Gebruers *et al*. [31] in soft wheat, some varieties tended to have consistently high or low AX contents. In order to visualize the relationships between the genotypic responses and the different environments explored, principal component analysis (PCA) was used. For each AX fraction the proportion of variance explained by the principal components was calculated (Figure 2). For TOAX and WUAX the first two principal components explained about 83% of total variation, whereas PC1 and PC2 together accounted for about 69% of the original variance of the WEAX variable and PC3 explained more than 17% of the variance. The PCA of varieties and environments for AX fractions were plotted on biplots. For TOAX and WUAX (Figures 3 and 4, respectively) all the environments fell onto the positive side of PC1, and only Jesi10 was located on the negative side for PC2. The varieties which were relatively stable across environments fell close to the PC2 axis; as an example (Figure 3), Saragolla and Tirex were below average for TOAX in all environments, the contents varied from 4.2 to 4.5% d.m and from 4.1 to 4.4% d.m., respectively. The varieties which fell on the right side of PC1 were the ones with a very strong interaction with the environment, presenting their max. potentiality of TOAX accumulation in Jesi10; in fact, for these varieties the range of the observed values was from 5.1 (Duilio) to 5.8% d.m. (Alemanno). The PCA biplot of WUAX fraction (Figure 4) again evidenced that some durum wheat varieties (Iride, Latinur, Tripudio, Dylan) had low absolute values for PC2: this fact generally indicated that the responses of these cultivars to the different environmental conditions were similar, their grain presenting low WUAX contents in most of their environments. As discussed for TOAX, the relative positions in the biplot of the varieties in positive side of PC1 reflected a significant interaction with the Jesi10 environment where they presented the highest WUAX values, in particular Alemanno (5.2% d.m.). Severo and Anco Marzio, which fell on the positive side of PC1 and PC2, generally evidenced WUAX contents slightly above the mean value; finally Trionfo, that had high positive values for PC1 and PC2, exhibited the highest WUAX content in Foggia10, its position on the biplot appearing in positive intersection with this environment. The biplot from PCA for WEAX fraction visually demonstrated that the interactions of variety with the environment were greater than for TOAX and WUAX and the durum wheat varieties likely responded to different environments in different ways (data not shown).

## 3. Experimental Section

### 3.1. Durum Wheat whole Grain Samples

For this study, a group of Italian commercial cultivars of durum wheat (*Triticum turgidum L. var durum*) was obtained from a set of agronomic trials carried out each year by the Research Unit for Cereal Quality of the Research in Agriculture Council (CRA-QCE). The trials are intended to evaluate yield and quality of durum wheat varieties grown in different sites under normal agronomic conditions. Thirty varieties were grown in two Italian areas adapt to durum wheat cultivation (Jesi, Central-Northern Italy, and Foggia, Southern Italy) in two consecutive years (2008–2009 and 2009–2010). For the comparison among different environments, nineteen out of the thirty varieties were considered. In each experiment, all cultivar were sown in 10 m^2^ plots in randomized blocks with three replications. Nitrogen fertilization was applied for both years according to local practice (about 150 and 90 U/ha, at Jesi and Foggia, respectively), with previous crop being fallow and leguminous in the Foggia and Jesi trials, respectively. The grain samples, blend of the three replicates, were milled to pass a 0.5 mm screen using a laboratory cyclone mill (Cyclotec 1093, FossItalia).

### 3.2. Environment Description

Jesi (altitude 97 m) presents a continental climate with a cold-humid winter and sultry-humid summer. Foggia (altitude 76 m) is characterized by a Mediterranean climate, a very warm summer and mild winter. The mean temperatures and total rainfall, referred at three seasonal periods, are reported in Figure 1. The means of minimal temperatures for the three considered periods were similar, whereas the maximum values of temperature presented differences from April to June between the two trial years, Foggia09 and Jesi09 having higher temperatures (mean values: 24.4 and 25.2 °C, respectively) in comparison with the successive year (mean values: 22.6 and 23.6 °C for Foggia10 and Jesi10, respectively). Anomalous precipitations occurred during the two trial years; moreover, total rainfall showed important differences between years and areas, with 616 and 749 mm at Jesi09 and Foggia09, respectively, and 702 (Jesi10) and 440 mm (Foggia10) in the successive year.

### 3.3. Grain Characteristics

A single kernel classification system (SKCS 4100, Perten Istruments, Sweden) was utilized for the grain hardness (expressed as hardness index), diameter and weight determination.

### 3.4. Chemical Analyses

All determinations were carried out in duplicate on two independent aliquots of each composite sample, blend of three replicates. The protein content was estimated with the nitrogen combustion method by using the Leco-FP 528 nitrogen analyzer [37]. Moisture content was determined at *T* = 120 °C with a thermo balance (Sartorius MA40-Göttingen, Germany). The arabinoxylans as total (TOAX) and unextractable (WUAX) content were analyzed by gas liquid chromatography (GLC). Following the treatment of Lai *et al*. [38] on rice, the cell wall material (CWM) was isolated according to the procedure of total (TDF) and insoluble (IDF) dietary fiber [39] by using an enzymatic kit for fiber determination (Bioquant, Merck, Germany). Briefly, 0.5 g of milled grains were incubated in phosphate buffer 0.08 M pH 6.00 with heat-stable α-amylase at 95–100 °C for 30 min, followed by cooling and hydrolysis with protease at 60 °C for 30 min at pH 7.5. Successively, at the same temperature, the last enzymatic step with amyloglucosidase at pH 4.5 was performed. For TOAX fraction analysis, the polysaccharide material was further precipitated with 95% ethanol (*T* = 60 °C) and then filtrated with the Fibertec semiautomatic system (FossItalia); this procedure found the CWM to contain 2.4–2.8% of proteins. The residue for WUAX determination was isolated by directly filtrating after the treatment with amyloglucosidase. Both residues were oven dried at T = 105 °C overnight, and, then, quantitatively recovered and hydrolysated with 10 mL of 2M TFA at *T* = 121 °C for two hours in a screw-capped glass tubes. Total neutral sugars were estimated by GLC [40,41], by using the following standard sugar mixture: D(−)arabinose, D(+)xylose, D-(+)mannose, D(+)glucose monohydrate (more than 99% Fluka), D-(+)galactose (more than 99% Sigma Aldrich) and internal standard β-D-allose (99%Acros Organics or 98%Alfa Aesar).

The chromatographic conditions employed were: column temperature 220 °C; injection and detector temperature 275 °C, helium flow rate 1.5 mL min^−1^. The instrument (Clarus 600, PerkinElmer, Shelton, USA) was equipped with an autoinjector and a Restek capillary column (RTX 2330 30 m, 0.32 mm, 0.2 μm). The AX and WUAX contents were calculated as in Ingelbrecht *et al*. [42]: AX = [%xylose + %arabinose − (0.7 × %galactose)] × 0.88. The soluble AX was calculated by difference: TOAX–WUAX.

### 3.5. Statistical Analysis

As reported by Li *et al*. [22] two independent aliquots of milled whole grains were considered statistical replicates of each grain composite sample. Analysis of variance (ANOVA) was performed with MSTATC program (Michigan State University, East Lausing, MI) by using a factorial model (mod.9) with cultivars, environment (locality and year) and variety by environment interaction; variety means were separated using Duncan’s multiple range test (*p* ≤ 0.01) for each environment separately and by combining the results across environments. Principal component analysis (PCA), performed with MATLAB software (R2010a version, MathWorks Inc., USA), was used to study the variations associated with genotype and environment for AX fractions. Simple correlation coefficients were also calculated.

## 4. Conclusions

The aim of this paper was to present the characterization of the grain of a wide range of durum wheat Italian varieties for the content in arabinoxylans, the most abundant polysaccharide constituents of the cell wall in wheat. Through an analytical procedure, AX contents were quantified in cell wall material isolated following total and insoluble dietary fiber method. Significant variations in the AX fractions were observed, with TOAX and WUAX varying from 4.1 to 5.8% d.m. and from 3.3 to 5.2% d.m., respectively. By statistical analysis, genotype and environment appeared to be important sources of variation for AX in the whole grain and significant genotype x environment interactions were also found. The contribution of this interaction to the total variability was found to be considerably lower than that of genotype or environment. Principal component analysis biplots allowed us to evidence some Italian durum wheat varieties that were consistently higher in AX fraction contents and stable across environments. Moreover, the range of different environmental conditions recorded in this study allowed us to evidence the environmental contribution to AX variability. Among the considered durum wheat varieties, Trionfo variety presented TOAX and WUAX contents higher than the mean values across all the environments. In addition, most varieties appeared to accumulate the highest TOAX contents in the environments characterized by the highest amounts of rainfall. Based on the indications from this study, the environment seemed to play an important role in WEAX variability and the performance of some genotypes appeared to be strongly associated with the grown environment. Finally, the present results provided useful information on the factors affecting the accumulation in the durum wheat grain of the arabinoxylan compounds. In addition, some varieties were identified in order to extend the research for defining suitable process conditions for the development of products with a physiological potential such as high fiber durum wheat pasta and bread.

## Figures and Tables

**Table 1:** Grain physical characteristics and total arabinoxylans (TOAX), water unextractable arabinoxylans (WUAX) and water extractable arabinoxylans (WEAX), calculated by the difference, TOAX–WUAX (mean values, % d.m., and standard deviation) in thirty durum wheat varieties grown in four environments (locality and year).

Quality Traits	Environments			
	Foggia09	Foggia10	Jesi09	Jesi10
Grain weight (g)	44.7 ± 4.1	43.6 ± 3.3	47.2 ± 5.8	46.7 ± 3.6
Grain diameter (mm)	2.7 ± 0.1	2.7 ± 0.1	2.8 ± 0.3	2.8 ± 0.1
Grain Hardness index	82.7 ± 4.6	88.0 ± 5.1	88.7 ± 3.7	86.8 ± 3.6
				
Protein content	12.1 ± 0.82	12.11 ± 0.64	16.2 ± 0.79	14.7 ± 1.46
TOAX content	4.7 ± 0.33	4.6 ± 0,27	4.5 ± 0.31	4.8 ± 0.41
WUAX content	4.0 ± 0.29	4.0 ± 0.31	3.9 ± 0.29	4.2 ± 0.44
WEAX content	0.7 ± 0.31	0.6 ± 0.17	0.6 ± 0.23	0.6 ± 0.29

**Table 2:** Mean Square of variety, environment and their interaction for arabinoxylan fraction content (total, TOAX, water unextractable, WUAX, and water extractable, WEAX) in the examined 19 durum wheat varieties grown in four environments.

	D.F.	Arabinoxylan Fractions		
Source of Variation		TOAX	WUAX	WEAX
Varieties (A)	18	749.07 ***	994.84 ***	94.82 ***
Error	18	0.001	0.001	0.001
Environments (B)	3	781.96 ***	530.05 ***	120.11 ***
A × B	54	73.42 ***	77.24 ***	83.51 ***
Error	57	0.001	0.002	0.001

D.F.: Degrees of freedom;

*** *p* ≤ 0.001.

**Table 3:** Mean values (% d.m.), standard deviation and Duncan’s test (*p* ≤ 0.01) for arabinoxylan fractions (total, TOAX, water unextractable, WUAX and water extractable, WEAX, calculated by difference, TOAX–WUAX) in the 19 durum wheat varieties grown in four environments. Different letters in the same column indicated that the values are significant different.

	Arabinoxylan Fractions		
Varieties	TOAX	WUAX	WEAX
Normanno	4.6 ± 0.27 g	3.8 ± 0.21 j	0.7 ± 0.34 cd
Trionfo	5.2 ± 0.30 a	4.6 ± 0.43 a	0.6 ± 0.32 gh
Iride	4.6 ± 0.21 ghi	3.8 ± 0.13 kl	0.8 ± 0.13 bc
Tirex	4.3 ± 0.17 m	3.7 ± 0.35 m	0.6 ± 0.34 hi
Anco Marzio	4.6 ± 0.21 ghi	4.1 ± 0.12 e	0.5 ± 0.10 jk
Simeto	4.8 ± 0.38 d	4.2 ± 0.47 d	0.7 ± 0.19 ef
Severo	4.8 ± 0.20 d	4.1 ± 0.06 e	0.7 ± 0.22 de
Creso	4.9 ± 0.34 c	4.0 ± 0.08 gh	0.9 ± 0.38 a
Claudio	4.5 ± 0.20 j	3.9 ± 0.22 j	0.6 ± 0.22 fg
Ciccio	4.8 ± 0.23 d	4.0 ± 0.36 fg	0.8 ± 0.19 b
Alemanno	5.0 ± 0.52 b	4.5 ± 0.50 b	0.5 ± 0.12 ij
Arnacoris	4.7 ± 0.41 e	4.3 ± 0.55 c	0.5 ± 0.19 k
Saragolla	4.4 ± 0.10	l 3.9 ± 0.14jk	0.6 ± 0.17 ij
Tripudio	4.4 ± 0.16 k	3.9 ± 0.04 hi	0.5 ± 0.14 jk
Imhopet	4.5 ± 0.18 ij	3.9 ± 0.27 i	0.6 ± 0.23 fgh
Latinur	4.5 ± 0.23 ghij	3.8 ± 0.16 j	0.7 ± 0.18de
Duilio	4.6 ± 0.35 f	4.1 ± 0.18 ef	0.6 ± 0.19 hi
Minosse	4.4 ± 0.26	l 3.7 ± 0.19 m	0.7 ± 0.31 de
Dylan	4.5 ± 0.14 hij	3.8 ± 0.17 l	0.8 ± 0.12 bc
average	**4.6** ± 0.23	**4.0** ± 0.24	**0.7** ± 0.12
lsd *p* ≤ 0.01	0.046	0.046	0.046
